# The transmembrane domain and luminal C-terminal region independently support invariant chain trimerization and assembly with MHCII into nonamers

**DOI:** 10.1186/s12865-021-00444-6

**Published:** 2021-08-12

**Authors:** Maryse Cloutier, Jean-Simon Fortin, Jacques Thibodeau

**Affiliations:** Laboratoire d’Immunologie Moléculaire, Département de Microbiologie, Infectiologie et Immunologie, Faculté de Médecine, Université de Montréal, Succ Centre-Ville, CP 6128, Montréal, QC H3C 3J7 Canada

**Keywords:** Antigen presentation, MHCII, CD74, Nonamerization, Transmembrane domain, Trimerization domain, RXR

## Abstract

**Background:**

Invariant chain (CD74, Ii) is a multifunctional protein expressed in antigen presenting cells. It assists the ER exit of various cargos and serves as a receptor for the macrophage migration inhibitory factor. The newly translated Ii chains trimerize, a structural feature that is not readily understood in the context of its MHCII chaperoning function. Two segments of Ii, the luminal C-terminal region (TRIM) and the transmembrane domain (TM), have been shown to participate in the trimerization process but their relative importance and impact on the assembly with MHCII molecules remains debated. Here, we addressed the requirement of these domains in the trimerization of human Ii as well as in the oligomerization with MHCII molecules. We used site-directed mutagenesis to generate series of Ii and DR mutants. These were transiently transfected in HEK293T cells to test their cell surface expression and analyse their interactions by co-immunoprecipitations.

**Results:**

Our results showed that the TRIM domain is not essential for Ii trimerization nor for intracellular trafficking with MHCII molecules. We also gathered evidence that in the absence of TM, TRIM allows the formation of multi-subunit complexes with HLA-DR. Similarly, in the absence of TRIM, Ii can assemble into high-order structures with MHCII molecules.

**Conclusions:**

Altogether, our data show that trimerization of Ii through either TM or TRIM sustains nonameric complex formation with MHCII molecules.

**Supplementary Information:**

The online version contains supplementary material available at 10.1186/s12865-021-00444-6.

## Background

Ii is a non-polymorphic type II transmembrane glycoprotein [1, 2]. It is mainly expressed in APCs and was originally found associated with MHC class II (MHCII) molecules [3, 4]. Ii assists the folding of MHCII αβ heterodimers and blocks their peptide binding groove to prevent the premature capture of Ags [4, 5]. While its role in MHCII assembly and transport is well documented, studies in transfected cells and knockout mice demonstrated the relative cell type- and allele-dependent importance of Ii expression [6–9].

Four Ii isoforms have been described in humans [10, 11]. Iip33 and p41 (named according to their molecular weight) differ due to the differential splicing of exon 6b, which encodes an additional 64 aa luminal domain. Iip35 and p43 also arise from this alternative splicing but they differ from p33 and p41, respectively, by the use of an alternative upstream start codon [10, 11]. The additional N-terminal 16 aa found in p35 and p43 encompass a cytoplasmic di-arginine (RxR) ER retention motif and a PKC-phosphorylable serine [12–15]. In its native state, this serine is part of a sequence recognized by β-COP, a component of COPI vesicles which mediate retrograde transport of cargo proteins from the cis-Golgi to the ER [16]. However, phosphorylation of the serine triggers the association of 14-3-3β, which is part of a family of ubiquitous proteins that regulate various biological activities. It has been postulated that, binding of 14-3-3β to Iip35 prevents recognition by β-COP and allows forward transport past the cis-Golgi [16, 17]. From the trans-Golgi, the MHCII/Ii complex will reach the endocytic pathway, either directly or after a short transit at the plasma membrane [18–22]. Once in endosomes, Ii is sequentially degraded, leaving CLIP into the groove of MHCII. This complex is recognized by the non-classical HLA-DM, which catalyzes the exchange of CLIP for a high-affinity peptide [23, 24].

Best characterized as a MHCII chaperone, recent studies have revealed that Ii is also engaged in a number of other immune functions [25–27]. For example, Ii regulates the trafficking of additional proteins, such as CD70, CD1 and MHCI [28, 29]. Interestingly, Ii has important biological properties that appear to be independent of its chaperoning activities. Indeed, a pool of Ii is displayed at the plasma membrane (thereby its CD74 designation) and serves as the receptor for MIF, a function hijacked by *Helicobacter pylori* [30, 31]. In light of its multifunctional nature, structural analyses are ongoing and key functional domains of Ii have been exposed. However, its crystal structure has yet to be determined, the major hurdle probably residing in the flexible nature of the membrane-proximal region [32].

**Fig. 1:**
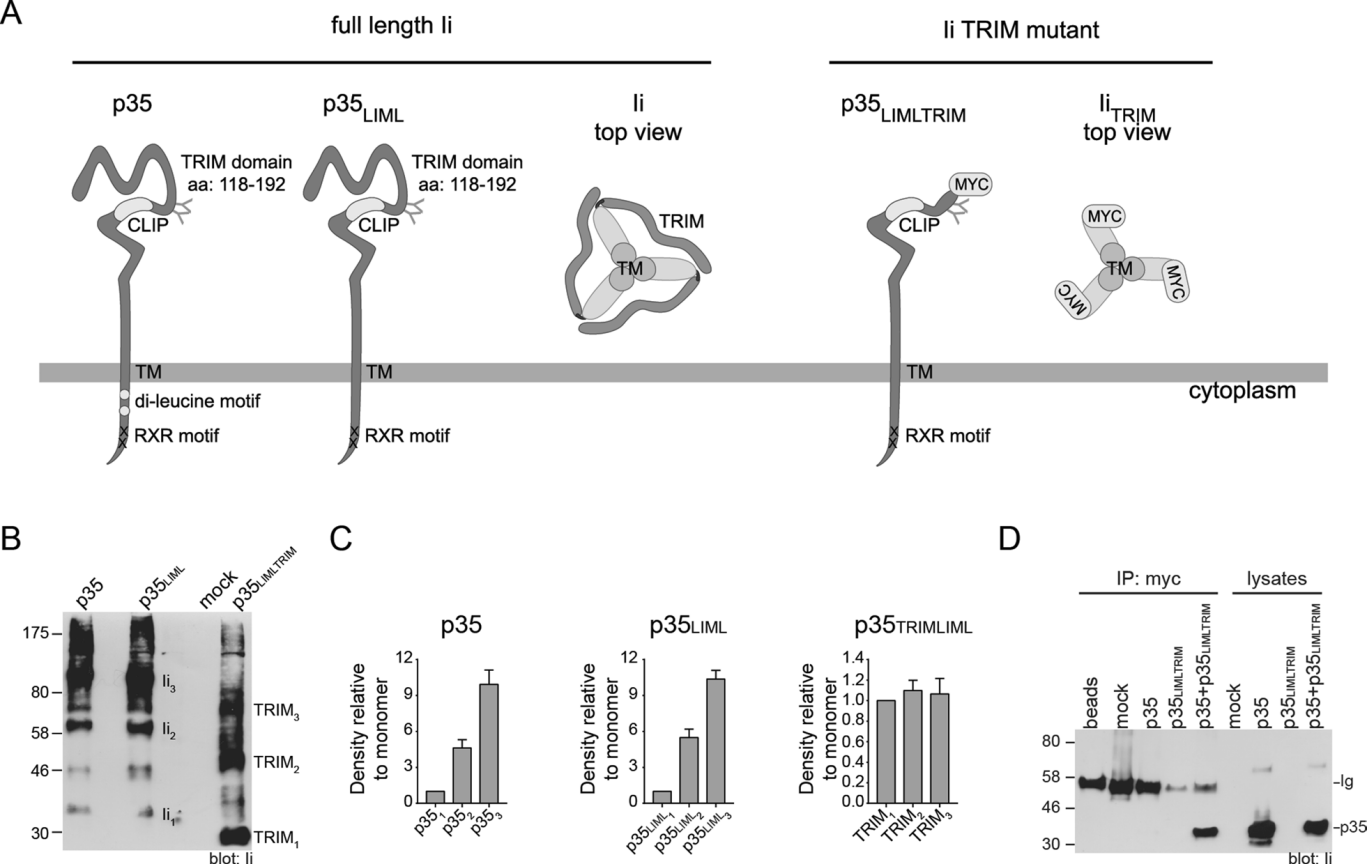
Formation of Ii trimers in absence of the TRIM domain. **a** Schematic representation of WT p35, p35_LIML_ and p35_LIMLTRIM_. The glycosylation sites, the CLIP region, the TM and the RxR motif have been illustrated on the different p35 molecules. Leucine-based endosomal targeting motifs (di-leucine; open circles in p35) were mutated to alanines in p35_LIML_ and p35_LIMLTRIM_. α-helices responsible for the C-terminal trimerization (TRIM domain) were deleted (Δ aa128 to 216 of p33) in the p35_LIMLTRIM_ mutant. Loss of the BU45 mAb epitope is coped with by addition of a myc/His-tag (detection 9e10 mAb). The Ii top view highlights how the trimerization domains oligomerize. The top view of the Ii_TRIM_ mutant shows the myc tag and the trimerization via the TM domains (circles) of Ii moieties. **b** HEK293T cells were transiently transfected with empty (mock) plasmid, p35, p35_LIML_ or p35_LIMLTRIM_. Cells were lysed in presence of reducible cross-linker DSP (400 µg/ml). Proteins were separated by SDS-PAGE in non-reducing conditions. Samples were blotted using a rabbit anti-CLIP serum. Monomeric (Ii_1_, TRIM_1_), dimeric (Ii_2_, TRIM_2_) and trimeric (Ii_3,_ TRIM_3_) forms are indicated on the right. Full-length blot is presented in Additional file 1: Figure 1A. **c** Histograms depicts the densitometry of Western blot bands (trimers and dimers) in relation to bands associated with monomers for each molecule. The densitometry was performed on blots from 3 independent experiments, as shown in (**b**). **d** HEK293T cells were transiently transfected with an empty plasmid (mock) or plasmids expressing p35, p35_LIMLTRIM_ or p35 and p35_LIMLTRIM_. Cells were lysed and immunoprecipitated using 9e10. Immunoprecipitated material and cells lysates were analyzed by SDS-PAGE and immunoblotted for WT p35 using Pin.1. Ig identifies antibody heavy chain detected in lanes with beads and IP products. Full-length blot is presented in Additional file 1: Figure 1B

Once translated and translocated into the ER, Ii rapidly trimerizes [33–35]. The structural basis for such self-association has been studied in mice and humans. Three regions of Ii have been shown to independently associate into trimers. First, a trimeric domain (TRIM) of 27 kDa (aa 118–192 of human p33/p41 encoded by exon 6) is located in the luminal region, just C-terminal of the CLIP region (Fig. 1a). Biochemical and nuclear magnetic resonance (NMR) spectroscopy studies on the recombinant fragment have confirmed the capacity of the human TRIM to trimerize [36–38]. Second, infrared spectroscopy and deletion studies have demonstrated that the human TM (aa 30–55) can trimerize in the absence of TRIM [39, 40]. Accordingly, using biophysical and computational methods, Dixon et al., demonstrated trimerization of the mouse TM in isolation [41]. Third, the group of Bakke has used NMR spectroscopy to demonstrate that a synthetic peptide, corresponding to the first N-terminal cytoplasmic 27 aa of hIip33, forms, in solution, an almost coplanar triple-stranded α-helical bundle in which two helices are parallel and one antiparallel [42].

While there is ample experimental and computational evidence that these different regions of Ii can trimerize, their relative importance remains debated. For one, the cytoplasmic region is not believed to play a role in self-association of full-length Ii because of its antiparallel nature. Rather, trimerization of this domain was proposed to facilitate sorting and promote endosomal retention as well as the generation of large endosomes [42, 43]. While the TM clearly self-associates, many groups have shown that it is not essential for trimerization to occur. Also, depending on the experimental system used, its deletion can slightly affect the association with HLA molecules [34, 37, 44, 45]. On the contrary, other experimental evidences point to the indispensable nature of TRIM for mouse or human Ii trimerization [34, 36, 44, 46]. Nevertheless, the TRIM-less mouse Iip10 proteolytic product found in endosomes has been shown to remain trimeric [47]. Importantly, these p10/p12 polypeptides of mice and humans are not only trimeric, they were also shown to remain associated with MHCII molecules as part of a nonameric structure [35, 47]. Thus, the relative roles of TM and TRIM in trimerization and the formation of high order structures with MHCIIs remain controversial. In humans, no study has yet concluded that TM is required, nor that TRIM is dispensable for Ii trimerization in the ER. While some data point to interactions between MHCII and both the TM and TRIM domains, their importance for the folding and nonamer formation remains to be fully characterized [37, 39].

Here, we have revisited these issues using a cellular system that allows assessing the capacity of Ii to trimerize and to associate into high-order structures with MHCIIs. Our data demonstrate that neither TM nor TRIM are essential for hIi trimerization. In addition, we show that any of these domains is sufficient to trigger the assembly into nonameric structures with MHCIIs, as long as the Ii moieties involved share the same domain. The importance of these trimerization regions for Ag presentation by MHCIIs and for Ii functions in general are discussed.

## Results

### Formation of Ii trimers in absence of the TRIM domain

Conflicting data exist in the literature regarding the importance of the TRIM motif of Ii in trimer formation as well as its relevance in the trafficking of MHCII-Ii complex [39, 41, 44, 46]. First, we asked if deletion of TRIM could affect the formation of hIi trimers in living cells. To address this question, we used a truncated version of p35 that preserves its glycosylation sites but lacks the three C-terminal α-helices forming the TRIM domain (Fig. 1a) [37, 38]. HEK293T cells were transiently transfected with either the wild-type (WT) p35, p35 lacking its endosomal sorting signals (p35_LIML_) or p35 lacking the TRIM motif and the sorting signals (p35_LIMLTRIM_). Mutation of the two leucine-based endosomal localization motifs favors the accumulation of Ii at the plasma membrane in the presence of MHCII molecules, thus providing a simple, indirect flow cytometry readout for ER egress [48–50]. It is important to stress that despite lacking strong sorting motifs, cell surface Ii_LIML_ and the Ii_LIML_/MHCII complex are nevertheless internalized (Additional file 1: Figure 1) in endosomes, where Ii gets degraded [51, 52]. When associated with MHCIIs, this passage of Ii into endosomes results in the formation of MHCII/CLIP complexes. In the absence of HLA-DM, these complexes are recycled to the plasma membrane and can be detected using a CLIP-specific mAb. For the flow cytometry detection of p35_LIMLTRIM_, which has lost both its luminal and cytoplasmic epitopes recognized by the BU45 and Pin.1 mAb, respectively, a myc tag was introduced at the C-terminal end (Fig. 1a).

The capacity of these individual molecules to homotrimerize was tested in transfected cells treated with the crosslinking agent DSP. After cell lysis, proteins were analyzed by WB using a polyclonal rabbit Ab recognizing the CLIP core sequence common to all constructions. p35 and p35_LIML_ were detected at various molecular weights, corresponding to monomers but mostly dimers and trimers (Fig. 1b, c). Interestingly, while p35_LIMLTRIM_ also formed dimers and trimers, we noted that a substantial amount of monomers remained in these conditions. A densitometric analysis of three independent experiments suggests that in the absence of TRIM, the formation of trimers is less efficient. While the proportions of dimers, which were shown to be disulfide-linked [53], appear to be independent of TRIM (Fig. 1b), the possibility remains that trimers forming in the absence of TRIM dissociate more easily upon cell lysis than WT Ii. This is in line with a previous report from Dixon et al., who observed different ratios of monomers to trimers for the Ii transmembrane depending on the detergent used for lysis [41]. Since the TM trimerization occurs within the membrane, disturbing its integrity affects the likelihood of observing high proportion of trimers. Interestingly, Dixon et al., did not see any dimers for the Ii transmembrane alone. To confirm that p35_LIMLTRIM_ can form trimeric complexes in the absence of crosslinking reagent, we tested by co-IP its ability to associate with WT Ii. HEK293T cells were transiently transfected with either p35, p35_LIMLTRIM_ or both p35 and p35_LIMLTRIM_. Cells were lysed and the TRIM mutant was immunoprecipitated using the 9e10 mAb against the myc tag (Fig. 1d). This mAb did not bring down p35 unless the p35_LIMLTRIM_ molecule was co-expressed, in line with the above-described results of crosslinking experiments showing dimers and trimers of p35_LIMLTRIM_ (Fig. 1b). Altogether, our data suggest that the TM is sufficient to allow the trimerization of hIi.

### Ii’s TRIM motif is not necessary for binding to MHCII molecules and to egress the ER

**Fig. 2:**
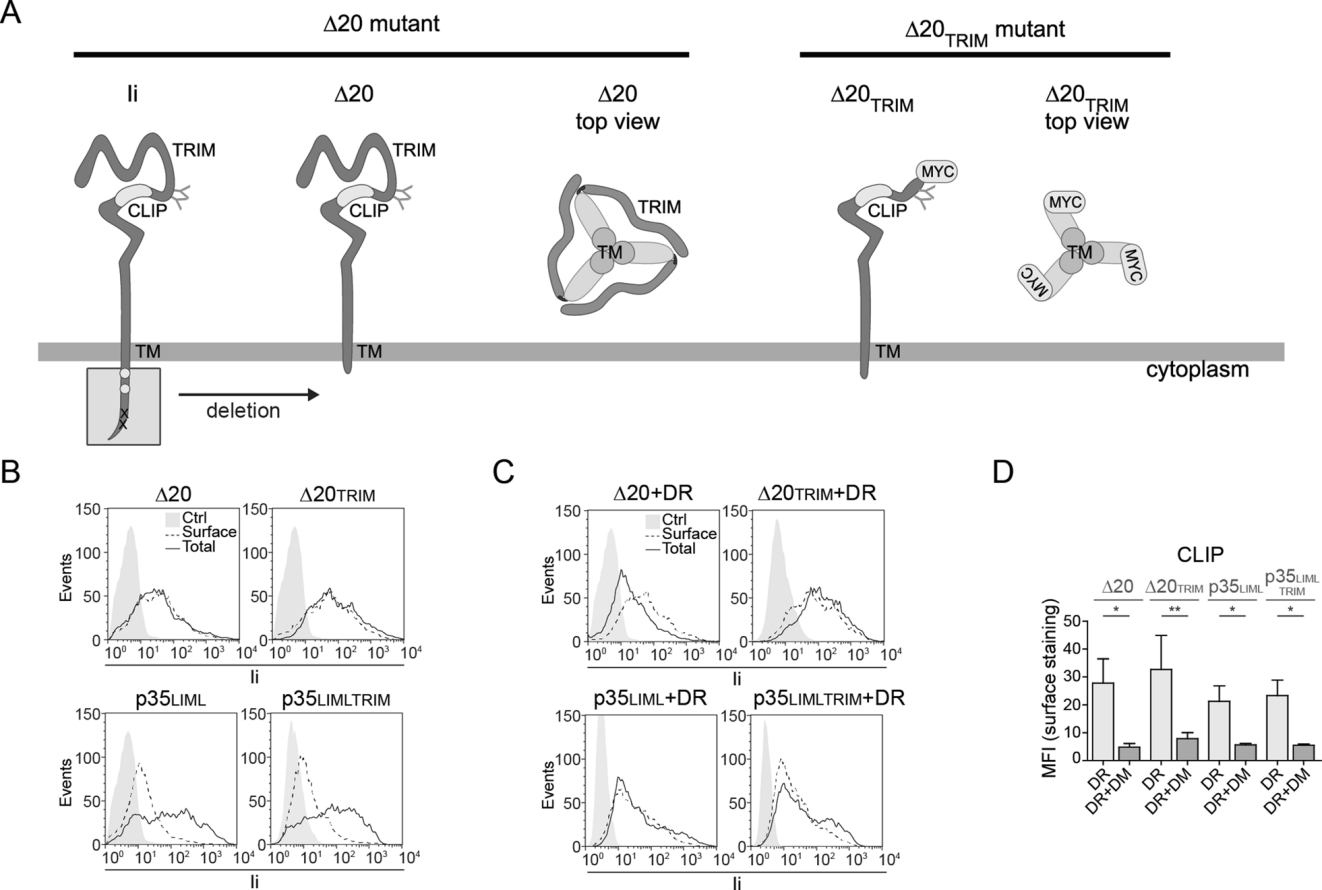
Ii C-terminal TRIM is not necessary for binding to MHCIIs and to egress the ER. **a** Schematic representation of Δ20 and Δ20_TRIM_. The lumenal glycosylation sites, CLIP region and TRIM are illustrated on the Ii molecule. Di-leucine endosomal targeting motifs (open circles) were removed following deletion of the first N-terminal cytoplasmic 20 aa (gray box) of p33 in Δ20 and Δ20_TRIM_ mutants. As for p35_LIMLTRIM_, the TRIM domain was deleted (Δ aa128 to 216 of p33) in the Δ20_TRIM_ mutant and a myc/His-tag was added (detection 9e10 mAb). The Ii top view highlights how the trimerization domains oligomerize. The top view of Δ20_TRIM_ mutant shows the myc tag and trimerization via the TM (grey circles). **b** Ii Δ20, Δ20_TRIM_, p35_LIML_ or p35_LIMLTRIM_ were transiently transfected in HEK293T cells. After 48 h, cells were stained to detect surface (black dotted line) and total (black line) Ii using mAbs BU45 or 9e10 (for Ii_TRIM_ mutants). **c** Cells were transfected as above together with DR and stained for surface and total Ii. **d** Cells from (**c**) and cells transfected as in (**c**) together with DM were stained for surface CLIP using CerCLIP.1 Ab. CLIP MFIs (mean fluorescence intensity) are expressed in a bar-chart. Error bars indicate the SD from at least five independent experiments. Student t-tests were performed; *p ≤ 0.001 and **p ≤ 0.05

We next asked whether deletion of TRIM could prevent the interaction of Ii with MHCII. As p35 does not exit the ER on its own, we tested the capacity of DR to assist surface expression of p35 and p35_LIMLTRIM_. As controls, we used Ii mutants devoid of their cytoplasmic tail (Δ20) and TRIM domain (Δ20_TRIM_) (Fig. 2a). These constructs were separately transiently expressed in HEK293T cells alone (Fig. 2b) or with DR (Fig. 2c). Cells were stained for the presence of Ii at the plasma membrane (surface) using BU45 (Fig. 2b, c, left panels) or 9e10 (Fig. 2b, c, right panels) mAbs. A fraction of the cells was permeabilized (total) before staining to ascertain expression of the Ii protein in conditions where surface expression was negative. The results clearly show that in the absence of DR, only the Δ20 constructs were gaining access to the plasma membrane. The Ii proteins that include a RxR motif are prevented from ER egress. However, DR rescued expression at the cell surface of all p35-based proteins, independent of the presence of TRIM. These results demonstrate that the TRIM domain is not required for Ii to associate with MHCII molecules.

Next, we ascertained that the Ii-MHCII interaction was genuine in the absence of TRIM and that the complex could interact with DM. While Ii can bind different regions of MHCII molecules [35, 37, 54, 55], the groove of DR is a major binding site that accommodates the CLIP_89–101_ region, just like any other nominal Ag [56]. Indeed, cell surface staining with the CerCLIP.1 mAb revealed the presence of CLIP at the cell surface (Fig. 2d). Interestingly, upon co-transfection of DM, CLIP was efficiently removed. These results show that in the absence of TRIM, both truncated p33 and p35 can still form trimers. When loaded with MHCII, they egress the ER and serve as substrates for lysosomal degradative enzymes that generate CLIP.

### No region other than TM or TRIM can support the formation of high-order complexes

**Fig. 3:**
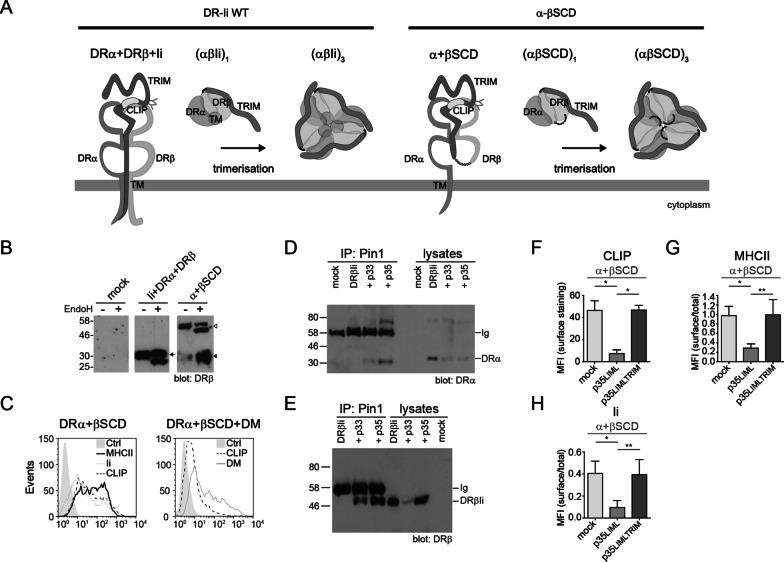
Trimerization through TRIM is sufficient for the formation of high-order complexes. **a** Schematic representation of WT DRα + DRβ/Ii and DRα + βSCD. Top view highlights αβ interaction with the CLIP domain. Top view of an Ii_3_ with αβ dimers illustrates a nonamer (αβIi)_3_. In βSCD, Ii and DRβ luminal domains are linked by a flexible gly_3_/ser/gly_3_ linker. Top views show association of DRα to βSCD and trimerization of the complex via TRIM. **b** HEK293T were mock-transfected or transfected with DRα + DRβ + Ii or with DRα + βSCD. Cell lysates, with or without EndoH, were analysed by WB and blotted for DRβ (XD5 mAb). Arrowhead represents EndoH resistant WT DRβ. Open arrowhead and arrowhead represent βSCD and a cleavage product of βSCD, respectively, both resistant to EndoH. **c** DRα + βSCD were transiently transfected in HEK293T. After 48 h, cells were stained to detect MHCII, Ii and CLIP (L243, BU45 and CerCLIP.1 mAbs, respectively) (left histogram). Cells were transfected as above as well as with DM and stained for surface CLIP or permeabilized to detect DM (Map.DM1 mAb) (right histogram). **d** Cells were either mock-transfected, transfected with DRα + βSCD alone or with p33 or p35. Cell lysates (right lanes) and material precipitated with the Ii-specific Pin.1 mAb (left lanes) were analyzed by WB. DRα was detected using the DA6.147 mAb. H chains of Ig used for IPs are indicated. **e** Samples from (**d**) were analyzed using the XD5.117 mAb specific for the DRβ1 extracellular domain. In **e, d,** Ig identifies antibody heavy chain detected in lanes with IP products. **f**–**h** Cells were transfected with DRα + βSCD alone or with p35_LIML_ or p35_LIMLTRIM_. **f** Cell surface MFI for CLIP (CerCLIP.1 mAb) are represented in bar charts. **g**, **h** MFI ratios obtained for surface versus total expression of MHCII (using L243) and Ii (using BU45) are represented in bar charts. Error bars indicate the SD from five independent experiments. Student t-tests were performed; *p ≤ 0.001 and **p ≤ 0.05. Full-length blots from this figure are presented in Additional file 1: Figure 1C–E

The above-described experiments demonstrate that the TM region of hIi can support the formation of trimers. Next, we confirmed the importance of TM using a different experimental system where the luminal β chain domains were covalently linked to the extracellular region of Ii, thereby eliminating transmembrane anchors (Fig. 3a). This linkage is possible because DRβ and Ii are type I and II proteins, respectively [57]. This single chain dimer (SCD) construct, when co-expressed with DRα, allows us to study the impact of different regions of a co-expressed Ii. Thus, we postulated that while p35 would retain this pseudo MHCII/Ii complex (DRα + βSCD), a TRIM-less p35 variant unable to associate with the Ii moiety of the SCD would have no impact on intracellular sorting.

First, we characterized the intracellular trafficking of βSCD. The covalent linkage of Ii and DRβ chain may prevent the problems encountered in a previous study where a TM-deleted form of Ii showed altered binding to MHCIIs [34]. When co-expressed with DRα, WB analysis of cell lysates demonstrate that a fraction of the recombinant βSCD protein becomes EndoH resistant (Fig. 3b, open arrowhead). Interestingly, the anti-DRβ chain-specific mAb also detected a fully EndoH-resistant fragment (filled arrowhead) migrating slightly faster than the WT DRβ chain (arrow) (Fig. 3b). This fragment most likely represents the DRβ moiety of the SCD that remains following the degradation of Ii in endosomes. These observations suggest that the SCD is properly folded, exits the ER and crosses the Golgi en route to the endosomes where Ii is degraded. Indeed, Fig. 3c shows that this chimeric protein is well expressed at the plasma membrane and ultimately generates CLIP/MHCII complexes (Fig. 3c, left panel), which serve as substrates for DM (Fig. 3c, right panel).

Then, the DRα + βSCD molecule was co-expressed with either p33 or p35. These WT Ii isoforms can form heterotrimers with the Ii moiety of the βSCD. Indeed, IP of the full-length Ii isoforms with the cytoplasmic tail-specific Pin.1 mAb showed the presence of both WT DRα and the recombinant βSCD, the latter being detected with the XD5 mAb directed at the β1 domain (Fig. 3d, e).

**Fig. 4:**
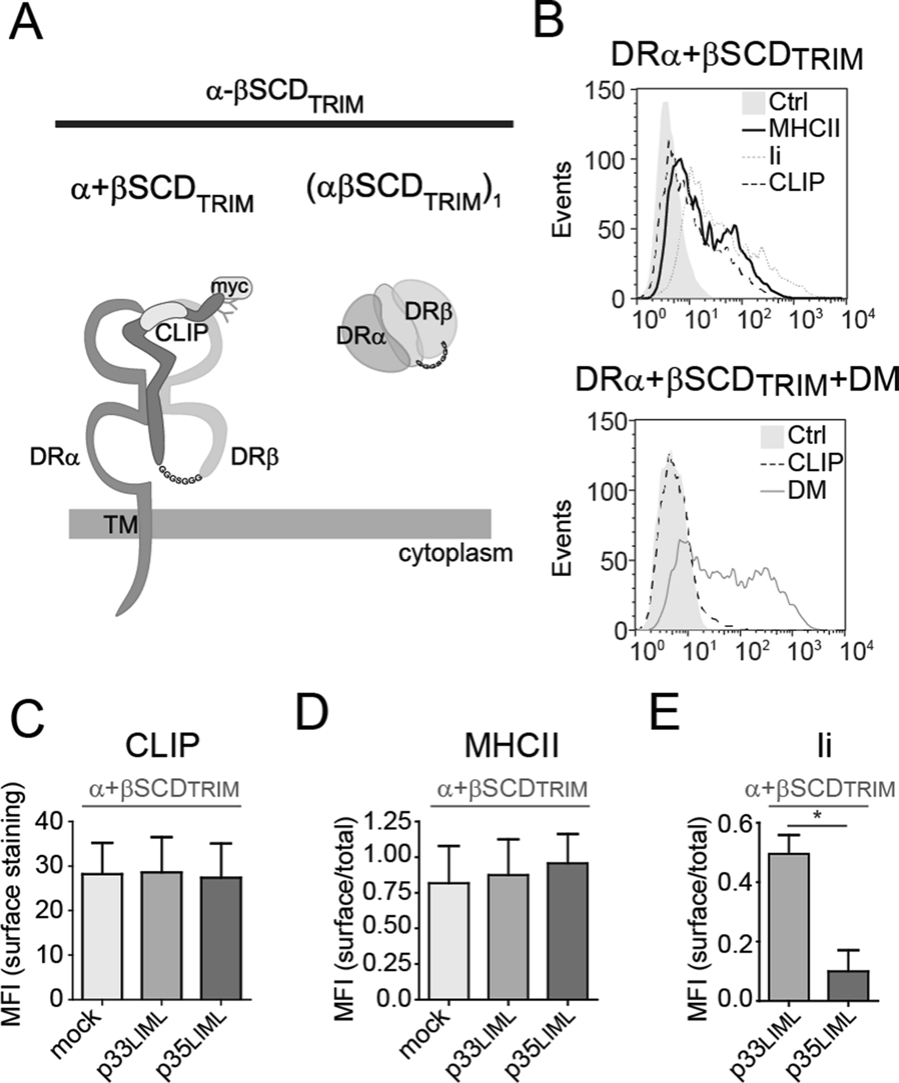
Lack of bidirectional TRIM interactions prevents formation pseudo-heptamers and/or pseudo-pentamers including βSCD. **a** Schematic representation of DRα and βSCD_TRIM_. In βSCD_TRIM_, the Ii_TRIM_ and DRβ luminal domains are linked by a flexible gly_3_/ser/gly_3_ linker. Top view on the right shows association of an α chain to the βSCD_TRIM_. **b** DRα and βSCD_TRIM_ were transiently expressed in HEK293T cells (upper histogram). After 48 h, cells were stained to detect MHCII, Ii and CLIP using L243, 9e10 and CerCLIP.1 mAbs, respectively. Cells were transfected as above, as well as with DM and stained for surface CLIP. A fraction of the cells was permeabilized to detect DM using Map.DM1 mAb (lower histogram). **c**–**e** Cells were transfected with DRα and βSCD_TRIM_ alone or together with p35_LIML_ or p35_LIMLTRIM_. After 48 h, cells were stained to detect surface CLIP (CerCLIP.1) (**c**). The surface over total expression ratio of MHCII (L243) (**d**) and Ii (BU45) (**e**) were calculated. Error bars indicate the SD from five independent experiments. Student t-tests were performed; *p ≤ 0.001

Interestingly, p35 prevents expression of DRα + βSCD at the plasma membrane, as shown by the absence of CLIP, MHCII and Ii on co-transfected cells (Fig. 3f–h, middle columns). This is due to the lack of a DRβ tail capable of masking the p35 ER retention motif [50, 58]. Importantly, a TRIM-less p35 could not prevent surface expression of DRα + βSCD, in line with the need for this domain in the interaction with the Ii moiety of βSCD (Fig. 3f–h, right columns). Finally, we repeated these experiments using WT p35 co-expressed with a βSCD devoid of its TRIM (Fig. 4a, b). Again, the lack of bidirectional TRIM-dominating interactions prevented the interaction between p35 and βSCD, as judged by the presence of the latter at the plasma membrane (Fig. 4c–e).

### The TRIM domain of Ii is not required for the formation of nonameric complexes

**Fig. 5:**
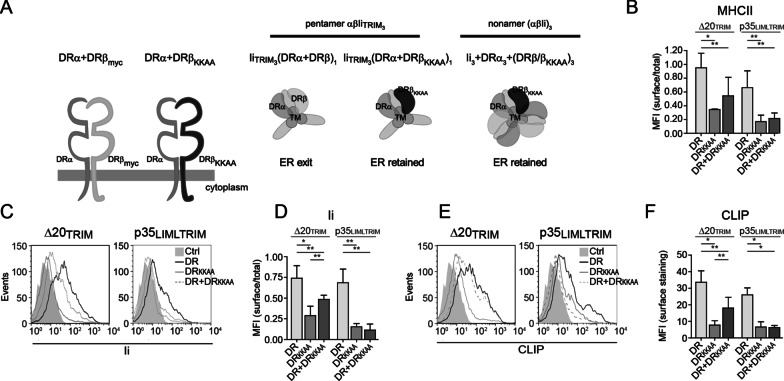
The TM domain supports formation of nonameric complexes in the absence of TRIM. **a** Left; schematic representation of DRα, DRβ_myc_ and DRβ_KKAA_. Right; illustration of the rational behind formation of pentamers and nonamers using the DRβ_KKAA_. If pentameric (DRαβ)_1_(Ii)_3_ complexes can egress the ER, the ER-retained (DRα + DRβ_KKAA_)_1_(Ii_TRIM_)_3_ complex will not impact WT (DRα + DRβ)_1_(Ii_TRIM_)_3_ complexes. In contrast, if formation of nonamers is the only outcome due to the expression of Iip35, the ER-retained DR_KKAA_ will trap a co-expressed WT DR that is incorporated in a same complex. **b**–**f** HEK293T cells were transiently transfected with DRα and DRβ_myc_ (DR) and/or DRα + DRβ_KKAA_ (DR_KKAA_) together Δ20_TRIM_ or p35_LIMLTRIM_. After 48 h, cells were analyzed by flow cytometry to evaluate surface to total expression ratio of MHCII using L243 (**b**). **c** Representative histograms showing surface expression of Ii and **d** bar graph shows surface to total expression ratio of Ii using BU45. **e** Representative histograms showing CLIP surface expression and **f** bar graph shows surface of CLIP using Cer-CLIP.1. Ctrl represent isotype control antibodies. **b**, **d**, **e** Error bars indicate the SD from at least three independent experiments. Student t-tests were performed; *p ≤ 0.001 and **p ≤ 0.05

We next investigated whether TRIM is required to assemble multiple MHC class II molecules around a multimeric Ii scaffold. For this, we designed an MHCII trap consisting of a mutant MHCII molecule (DR_KKAA_) bearing a stringent KKAA cytoplasmic ER retention motif (which cannot be overcome in any ways) (Fig. 5a, left panel) [49]. We asked whether a TRIM-less Ii, once bound to DR_KKAA_, could catch and prevent ER egress of other co-expressed WT MHCIIs, thereby confirming the formation of multimeric complexes comprising multiple Ii and MHCII molecules (Fig. 5a, right panels).

First, we tested the control Ii Δ20_TRIM_ variant, which is devoid of RxR and di-leucine cytoplasmic motifs. Transfected HEK cells were analysed for the expression of DR and Ii at the plasma membrane by flow cytometry (Fig. 5b–e). A fraction of the cells was also permeabilized to calculate the surface over total mean fluorescence intensity (MFI) ratio. This allows us to evaluate and to compare indirectly the efficiency of ER egress (Fig. 5b, d). When control DR was expressed as the sole source of MHCIIs, DR and IiΔ20_TRIM_ were both detected at the plasma membrane (Fig. 5b–d). Also, a substantial amount of DR/CLIP complexes were detected at the cell surface, confirming genuine association between MHCII and Ii (Fig. 5e, f). In contrast, when co-transfected with DR_KKAA_, very little Δ20_TRIM_ was able to make it to the plasma membrane (Fig. 5c, d) and, as those molecules that escaped retention by DR_KKAA_ (Fig. 5b) trafficked on their own, no CLIP/DR complexes could be detected (Fig. 5e, f). Interestingly, when the two DR were co-expressed with Δ20_TRIM_, we found some Ii and MHCII molecules at the plasma membrane (Fig. 5b–d). Also, the presence of CLIP demonstrates that DR/Δ20_TRIM_ complexes gained access to the endocytic pathway and thus were free of DR_KKAA_ (Fig. 5e, f) These findings suggest that in the presence of Δ20_TRIM,_ WT DR most likely assemble independently from DR_KKAA_, and can egress as pentamers (α_1_β_1_Δ20_TRIM__3_), or even trimers (α_1_β_1_Δ20_TRIM1_). Thus, the use of control IiΔ20_TRIM_ could not inform on the capacity of Ii_TRIM_ to assemble different MHCIIs into the same complex.

We then tested the impact of Iip35_LIMLTRIM_ in cells expressing DR. Our results show that DR was found at the plasma membrane together with Ii and CLIP (Fig. 5b–f). Again, this does formally demonstrate the formation of Ii trimers or trafficking of the complex in the form of a nonamer. As expected, DR_KKAA_ could not rescue the ER egress of Iip35_LIMLTRIM_ as both molecules have retention motifs. Accordingly, no CLIP was present at the cell surface (Fig. 5e, f). Interestingly, when p35_LIMLTRIM_ was expressed with both DR and DR_KKAA_, class II, Ii and CLIP were not found at the cell surface (Fig. 5c–f). This is in stark contrast with the results obtained above using IiΔ20_TRIM_. This result is in line with a model where Iip35_LIMLTRIM_ does trimerize in the ER and stochastically associates with DR and DR_KKAA_. As it is likely that each and every p35_LIMLTRIM_ homotrimer recruited at least one DR_KKAA_ molecule, this prevented surface expression of all MHCII species. Altogether, these results confirm that TRIM is not required for Ii and MHC II molecules to associate into multimeric structures.

### The TRIM supports the scaffolding of nonamers in the absence of Ii’s TM

**Fig. 6:**
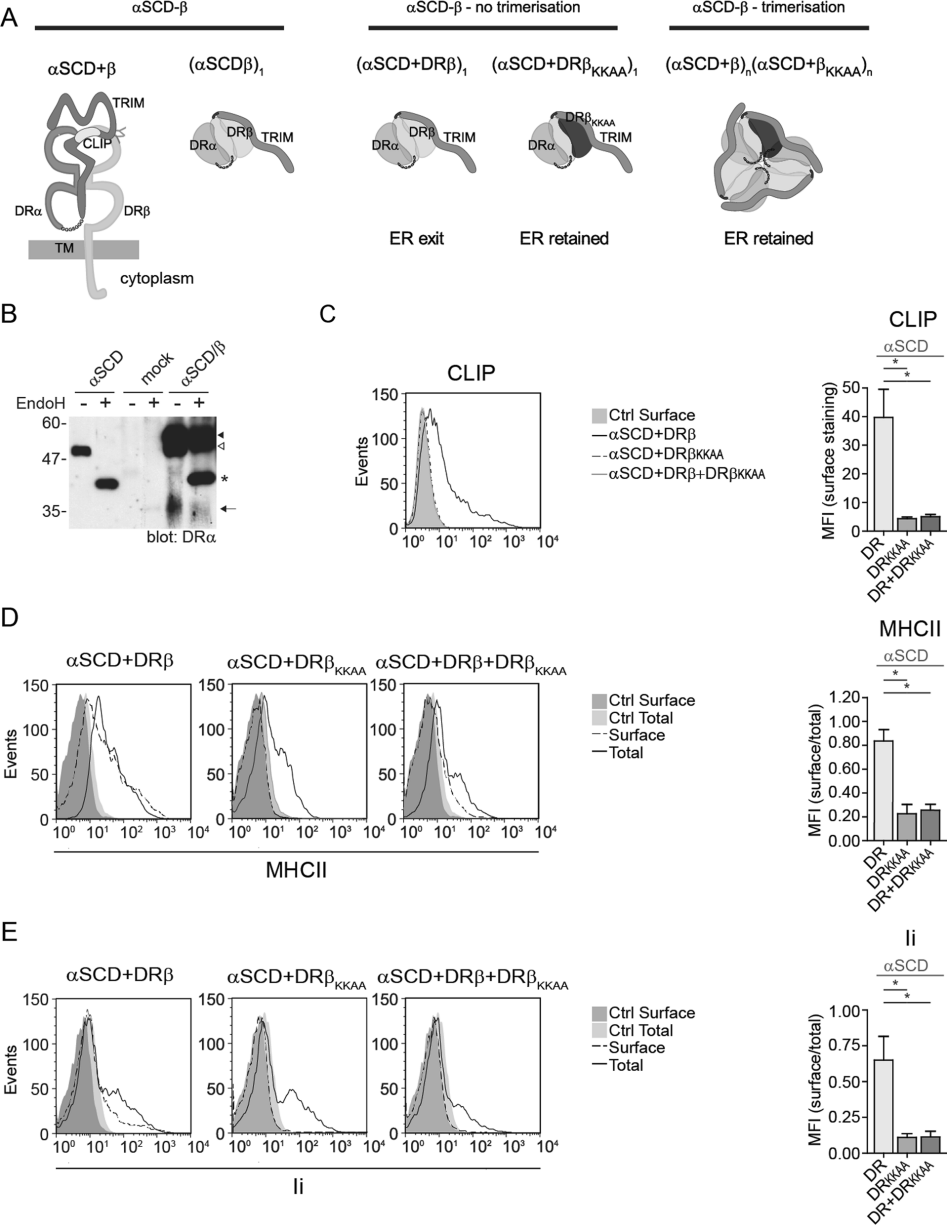
DR is retained by DR_KKAA_ upon formation of nonameric-like structures. **a** Schematic representation of αSCD and DRβ and illustration of the rationale behind the use of αSCD with DRβ_KKAA_. Left; DRα and Ii luminal domains are linked by a flexible gly_3_/ser/gly_3_ linker (αSCD). Top view shows the association of αSCD with a β chain. Middle; when co-expressed with DRβ_KKAA_, DRβ will still egress the ER if αSCD does not trimerize via the TRIM domain. Right; formation of a trimer through TRIM will force the incorporation of both DRβ and DRβ_KKAA_ in the same complex, which will be ER retained. **b** HEK293T cells were mock-transfected or transfected with αSCD or with αSCD and DRβ. Cell lysates were treated with or without EndoH and blotted for DRα (DA6.147). Open arrowhead and arrowhead represent EndoH resistant forms of αSCD with different types of complex sugars. Star represents the EndoH sensitive αSCD. Arrow represents cleavage products of αSCD. Full-length blot is showed in Additional file 1: Figure 1F. **c**–**e** HEK293T cells were transiently transfected with DRβ_myc_ (DR) and/or DRβ_KKAA_ together with αSCD. After 48 h, cells were analyzed by flow cytometry to evaluate CLIP surface expression, using CerCLIP.1 (**c**), MHCII (**d**) and Ii (**e**) surface over total expression ratio using L243 and BU45, respectively. Representative histograms of CLIP surface expression (**c**), MHCII surface and total expression (**d**) and Ii surface and total expression (**e**) are shown. Ctrl represent isotype control antibodies. Error bars indicate the SD from at least three independent experiments. Student t-tests were performed; *p ≤ 0.001 and **p ≤ 0.05

Experiments using truncated soluble molecules have demonstrated the rapid trimerization of Ii and the subsequent formation of complexes of variable stoichiometry with MHCIIs [45]. We have addressed in transfected cells the impact of deleting Ii’s N-terminal region, including TM, on the assembly with MHCIIs. To ascertain the efficient binding of the CLIP region into the peptide-binding groove of DR, Ii was covalently linked to the extracellular portion of DRα (αSCD), as previously described (Fig. 6a) [50]. When expressed on its own in HEK293T cells, the αSCD remains EndoH sensitive and is most likely trapped in the ER (Fig. 6b). As expected, when co-transfected with the membrane-anchored DRβ, the αSCD is strongly expressed and a large proportion becomes EndoH-resistant (arrowheads, Fig. 6b). Also, an EndoH-resistant degradation product was detected (arrow, Fig. 6b), in line with the ER/Golgi egress of the αSCD/β complex and the eventual endosomal processing of the Ii moiety. Accordingly, CLIP, Ii and DR were all detected at the plasma membrane by flow cytometry (Fig. 6c–e, left columns). However, when the αSCD was co-transfected with DRβ_KKAA_ chain instead of DRβ, the complex was not found at the cell surface (Fig. 6c–e). Interestingly, when αSCD, DRβ_KKAA_ and WT DRβ were all co-expressed, there was no CLIP, Ii or DR at the plasma membrane (Fig. 6c–e, right columns). The data are compatible with a model where the αSCD first trimerizes [59] and the stochastic incorporation of the available DRβ chains will result in the ER retention of most nonamer-like complexes by DRβ_KKAA_. We conclude that the TM domain of Ii is not a prerequisite for the assembly of multimeric structures comprising multiple MHCII molecules. These data are in agreement with those of Cresswell and collaborators showing that the proteinase K digestion of Ii in MHCII/Ii complexes generates a C-terminal K3 fragment, which includes TRIM and by itself can retain the complex in its nonameric conformation [35].

## Discussion

Newly translated full-length Ii chains swiftly trimerize upon translocation into the ER [33, 36]. The need for such self-association is unclear. Data accumulated so far, including those presented here, lead to the conclusion that two distinct regions, highly conserved and encoded by separate exons, can mediate self-recognition of Ii. Besides its chaperone function, free Ii has been shown to accumulate at the plasma membrane, principally in APCs [60, 61]. At least three different functions of this pool of cell surface Ii/CD74 have been characterized. First, Ii serves as a receptor for MIF [30]. While both the ligand and receptor are trimeric, modeling studies point to a possible dodecameric structure where each Ii moiety binds a MIF trimer [62]. Future studies should address the need for these interactions in the generation of a signaling platform, which includes CD44, capable of activating MAPK and to trigger production of pro-inflammatory cytokines [63]. Crosslinking of CD74 also leads to the intramembrane cleavage and the release of the intracellular domain (ICD) [64, 65]. This short domain enters the nucleus and modulates the transcriptome of APCs [66]. While peptides corresponding to the cytoplasmic domain of Ii has been shown to trimerize [42], the structural basis underlying the nuclear activity of the ICD is unknown. In the context of full length Ii, the presence of three cytoplasmic tail was shown to be essential to endosomal targeting and for shaping endosomes morphology [43, 52]. As this activity of Ii is thought to be important for Ag presentation, it may explain in part the need for trimerization [67]. Thus, it is likely that a multi-functional Ii requires multiple trimerization domains, including an extracellular one (TRIM) to rigidify an otherwise unstructured Ii membrane-proximal region and to create a MIF binding domain. While the exon 6b-encoded polypeptide is C-terminal to these trimerization sites, it does not appear to affect the overall stoichiometry [68]. However, the N-terminal extension of p35/p43 could modulate enlargement of endosomes or gene expression, two issues that will require further investigations. Also, the capacity of p35 to possibly interact specifically with COPII vesicles and fine tune ER egress remains to be addressed [50].

When considering the chaperone role of Ii, the need for trimerization in the context of MHCII transport is not readily apparent. Nonameric complexes (αβIi)_3_ were first described in the early 1990s and are a direct consequence of Ii’s ability to form trimers [33]. However, in 2011, it has been proposed that due to structural constraints, Ii/MHCII complexes can only exist as pentamers αβ(Ii)_3_ [69]. While our results confirmed that pentamers can to exit the ER, we have also clearly demonstrated that the ER retention motif of p35 promotes the formation of nonameric structures [49, 50]. Indeed, there must be a direct interaction between p35 and the MHCII to inactivate the ER retention signal, thus forcing the addition of αβ heterodimers until all RxR motifs are matched [63, 70]. Mice don’t express p35 and we must envisage that an alternative regulatory checkpoint predominates. Early work by the group of Cresswell had shown that calnexin remains bound to the complex until the Ii trimer is fully saturated with MHCIIs [71]. This mechanism may be more stringent in mice than in humans in preventing “premature” egress of pentamers and heptamers [72]. It is important to stress that in some experiments, we did not have direct or indirect evidence that Ii exited the ER as a trimer. For example, in Fig. 5, those complexes exiting to the plasma membrane could theoretically be formed over a dimer of IiΔ20_TRIM_. Indeed, we have shown in Fig. 1b that such dimers of Ii (Ii_2_) can be visualized on Western blots after crosslinking. Interestingly, these dimers have been described almost 40 years ago by Koch and Hammerling [53]. They were found to be disulfide-linked through the free intracytoplasmic cysteine residue near TM. Still, formation of these dimers or trimers is not mutually exclusive. Noteworthy, SCDs cannot form such dimers since they do not include the cytoplasmic cysteine of Ii.

Beyond the debate regarding the stoichiometry of the complexes leaving the ER, the need for multimerization of MHCII in Ag presentation remains nebulous. At one extreme, Ii was even shown to be dispensable for MHCII assembly/trafficking in some cell lines and knockout mice [6, 73, 74]. This is certainly non-physiological as MHCIIs and Ii are co-expressed and the latter is usually found in vast excess [75]. Few studies have addressed the importance of TRIM and TM in Ag presentation. Deletion experiments of either domain have produced variable results and stoichiometry of the resulting complexes has not always been thoroughly monitored. On one hand, Germain has shown that truncation of Ii after CLIP does not alter MHCII assembly, trafficking and peptide acquisition, suggesting that TRIM is not a prerequisite for Ag presentation [76]. However, this study did not address the trimeric nature of the truncated Ii. On the other hand, in mice, Koch and collaborators found that Ii oligomer formation through the C-terminal region is needed for HEL presentation [46].

No study has tackled the systematic comparison of Ag presentation efficiency using Iiαβ, Ii_3_αβ or (Iiαβ)_3_ complexes. The difficulty resides in our capacity to generate structurally comparable complexes of defined stoichiometry. In our recent studies, we made use of the αSCD and the results suggested that the TM of Ii is not required in living cells for the formation of Ii/MHCII complexes of variable stoichiometry. Here, we have confirmed these results and extended the conclusions to the TRIM of hIi. Also, we have shown that no region other than the TM or TRIM (or even MHCIIs themselves) promote Ii self-association. By using SCDs devoid of TRIM, we were able to compare the trafficking of Ii_1_α_1_β_1_ with WT nonameric Ii_3_α_3_β_3_ complexes. While we have not monitored Ag presentation per se, the capacity of all these constructs to generate MHCII/CLIP complexes and to interact with DM suggest that they are structurally and functionally similar. Interestingly, the group of Hirano has recently provided evidence that HLA-DPβ allotypes bearing a glycine at position 84 (DP^84Gly^), such as DP4, do not bind Ii through CLIP [77]. They further showed that this MHCII molecule cannot form nonamers and rather engages Ii in a Ii_1_(αβ)_1_ complex. While Ii chaperones DP4 to the endocytic pathway, more studies will be needed to determine if this peculiar stoichiometry intervenes in the association of these alleles with autoimmune diseases [78].

## Conclusion

In conclusion, the purpose of the two distinct trimerization domains of Ii in the chaperoning of MHCIIs remains an open question. As mentioned above, it is possible that the luminal TRIM serves some MHCII-independent functions and that the structural features required for Ii to chaperone other cargos are dependent on TRIM. Future structure–function studies addressing the interaction of Ii with other molecules, such as CD70 and possibly CD1d, should shed light on this issue [79–81].

## Methods

### Plasmids and mutagenesis

pBud DR, pBud DM, pcDNA3.1 DRα, pBud αSCD, pcDNA3.1 DRβ_myc_ and pcDNA3.1 DRβ_KKAA_, pcDNA3 Ii, pcDNA3 p33, pcDNA3 p33_LIML_, pcDNA3 p35, pcDNA3 p35_LIML_ and pBud Δ20 Ii have been described previously [48–50, 58, 82]. The β single-chain dimer (βSCD) linking the luminal domain of DRβ (aa 1–199) to Ii’s luminal region (aa 57–232) using a (Gly)_3_(Ser)_1_(Gly)_3_ linker was created has described for αSCD [50]. Mutants lacking the TRIM domain (aa 128–216 in p33) were created by PCR overlap extension for p35_LIML_, Δ20 and βSCD, giving rise to the pBud p35_LIMLTRIM_, pBud Δ20_TRIM_ and pBud βSCD_TRIM_, respectively.

### Abs, immunoprecipitation (IP) and Western blot (WB)

The following mouse mAbs were described previously [58, 82]: BU45 (C-terminal region of hIi); Pin.1 (cytoplasmic tail of hIi), L243 (HLA-DR); DA6.147 (cytoplasmic tail of DRα) XD5 (DRβ); CerCLIP.1 (CLIP); MaP.DM1 (DM); 9e10 (myc tag) (Biolegend, San Diego, CA) and the rabbit anti-CLIP (CLIP region of Ii) (a kind gift from Dr P. Cresswell, Yale University).

Alexa Fluor 488- or 633-coupled goat anti-mouse secondary Abs (Invitrogen, Burlington, ON) were used for flow cytometry. For WB, Peroxidase-AffiniPure goat anti-mouse IgG (H + L) and Peroxidase-AffiniPure goat anti-mouse Fc specific (Jackson Immunoresearch, West Grove, PA) were used. For IPs, cells were lysed at 4 °C in 1% Triton-X100. Lysates were analyzed as controls and all samples were subjected to reducing SDS-PAGE. Proteins on immunoblots were detected by chemiluminescence (Roche Applied Science, Laval, Qué.). For crosslinking experiments, cells were lysed in 1% Triton and 400 µg/mL DSP (dithiobis (succinimidyl propionate)) (Sigma Aldrich, St-Louis, MO). For Endo H resistance assays, total lysates were treated with Endo H (New England Biolabs), according to the manufacturer’s recommendations. Proteins were analyzed in non-reducing conditions by SDS-PAGE.

### Cell lines and flow cytometry

For transient expression, HEK293T cells were transfected using polyethyleneimine (Polyscience, Warrington, PA) and stained after 48 h. To determine surface expression, live cells were stained on ice and analyzed by flow cytometry on a FACSCalibur or FACSCantoII. To determine total expression of MHCII and Ii, cells were fixed in 4% paraformaldehyde, permeabilized, and stained, as described previously [82]. Forward and side scatter gating strategy was used to gate on single cells.

## Supplementary Information

**Additional file 1**.** Internalisation of Ii mutants lacking the endosomal targeting motif**. HEK293T cells were transiently transfected with DR+p33, DR+p33_LIML_ or αSCD/β. After 48h, cells were stained on ice with BU45. Cells were shifted to 37°C and aliquots were stained after 0, 15 and 30 minutes using a goat anti-mouse IgG shifted to 37oC and aliquots were stained after 0, 15 and 30 minutes using a goat anti-mouse IgG experiments and error bars indicate the standard deviation of triplicates. Paired Student’s t-tests were performed; *: p ≤ 0.05 and **: p ≤ 0.01.** Autoradiograms used to prepare figures in the paper**. A and B, see figure 1. C, D and E, see Fig. 3. F, see Fig. 6.

## Data Availability

The datasets used and/or analysed during the current study are available from the corresponding author on reasonable request.

## Abbreviations

αSCD: Alpha single-chain dimer
βCOP: Protein complex coatmer beta subunit
βSCD: Beta single chain dimer
DSP: Dithiobis (succinimidyl propionate)
ER: Endoplasmic reticulum
HEK: Human embryonic kidney cells
HEL: Hen egg-white lysozyme Ag
hIi: Human Ii
ICD: Intracellular domain
Ii: Invariant chain
IP: Immunoprecipitation
MFI: Mean fluorescence intensity
MHCI: MHC class I
MHCII: MHC class II
MIF: Macrophage inhibiting factor
NMR: Nuclear magnetic resonance
PCR: Polymerase chain reaction
PKC: Protein kinase C
TM: Transmembrane domain
TRIM: Trimerization domain
WB: Western blot
WT: Wild type
