# Supplementary material for: The transmembrane domain and luminal C-terminal region independently support invariant chain trimerization and assembly with MHCII into nonamers
Source: BMC Immunol. 2021 Aug 12;22:56. doi: 10.1186/s12865-021-00444-6 (PMC8362237; doi:10.1186/s12865-021-00444-6)
Supplement: Supplementary file 1 — Additional file 1. Internalisation of Ii mutants lacking the endosomal targeting motif. HEK293T cells were transiently transfected with DR+p33, DR+p33LIML or αSCD/β. After 48h, cells were stained on ice with BU45. Cells were shifted to 37°C and aliquots were stained after 0, 15 and 30 minutes using a goat anti-mouse IgG shifted to 37oC and aliquots were stained after 0, 15 and 30 minutes using a goat anti-mouse IgG experiments and error bars indicate the standard deviation of triplicates. Paired Student’s t-tests were performed; *: p ≤ 0.05 and **: p ≤ 0.01. Autoradiograms used to prepare figures in the paper. A and B, see figure 1. C, D and E, see Fig. 3. F, see Fig. 6. [file 12865_2021_444_MOESM1_ESM.pdf]

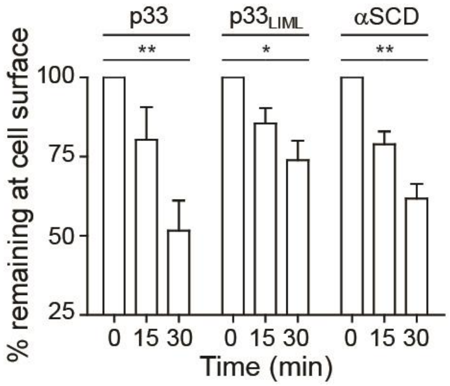

Internalisation of Ii mutants lacking the endosomal targeting motif. HEK293T cells were transiently transfected with DR+p33, DR+p33$_{LIML}$ or αSCD/β. After 48h, cells were stained on ice with BU45. Cells were shifted to 37°C and aliquots were stained after 0, 15 and 30 minutes using a goat anti-mouse IgG secondary antibody coupled to Alexa Fluor 488. The graph is representative of four independent experiments and error bars indicate the standard deviation of triplicates. Paired Student's t-tests were performed; *: $p \leq 0.05$ and **: $p \leq 0.01$

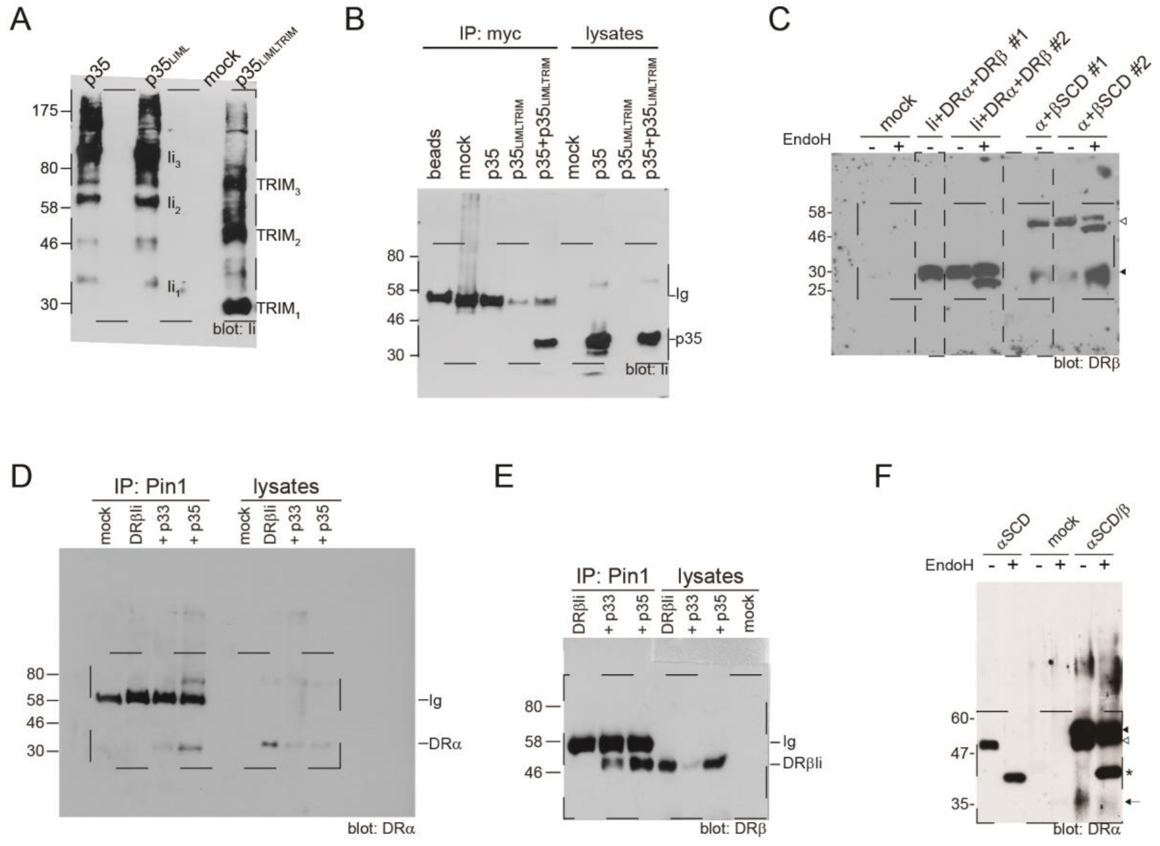

Autoradiograms used to prepare figures in the paper. A and B, see figure 1. C, D and E, see figure 3. F, see figure 6.
